# Editorial: Role of extracellular vesicles in promoting cancer stem cell properties, tumor aggressiveness and metastasis formation in solid tumors

**DOI:** 10.3389/fimmu.2024.1504676

**Published:** 2024-10-17

**Authors:** Giulia Bertolini, Orazio Fortunato, Serena Lucotti

**Affiliations:** ^1^ Epigenomics and Biomarker of Solid Tumors Unit, Fondazione IRCCS Istituto Nazionale dei Tumori, Milan, Italy; ^2^ Children’s Cancer and Blood Foundation Laboratories, Departments of Pediatrics, and Cell and Developmental Biology, Drukier Institute for Children’s Health, Meyer Cancer Center, Weill Cornell Medicine, New York, NY, United States

**Keywords:** extracellular vesicle (EV), metastasis (cancer metastasis), cancer stem cell (CSC), cancer, tumor aggressiveness

Cancer progression is a complex, multistep process involving the growth, invasion, and dissemination of primary tumor cells to distant organs (1). In recent years, growing evidence has shown that tumor cells and the tumor microenvironment, comprised of stroma and immune host cells, influence each other via exchange of extracellular vesicles (EVs) (2). The release of EVs by tumor, immune, and stromal cells within primary tumors has been implicated in most stages of tumor invasion and metastasis (3). The EV cargo, including nucleic acids, proteins, and lipids, contributes to the modulation of the cancer stem cell (CSC) properties of tumor cells, which sustain primary tumor maintenance, resistance to therapy and the metastatic to distant sites (4). Beyond their endogenous functions, EVs are currently being tested as delivery vehicles for various biomolecules (such as nucleic acids, lipids, proteins, and small molecule therapeutics) for local or systemic treatment (5). Due to their stability in the bloodstream, circulating EVs and their cargo also hold great potential as biomarkers for cancer diagnosis and therapeutic response (6).

This Research Topic covered several aspects of the role of EVs in modulating progression, dormancy, stemness, and metastasis in different solid tumors.

Featured studies have highlighted EV involvement in Neuroblastoma (NB) progression, including tumor growth, metastasis, and therapeutic resistance. NB is the most common extracranial solid tumor in children and a leading cause of cancer-related deaths in pediatric patients due to the quick development of resistance and relapse. The review by Dhamdhere et al. summarizes the recent findings on NB-EVs, exploring the therapeutic strategies for their targeting and discusses the use of EVs as biomarkers for improved diagnosis and treatment of NB.

The work of Pandya et al. explores how EV-mediated interactions between CSCs and the tumor microenvironment promote stemness, tumor progression, and metastasis in for Non-small Cell Lung Carcinoma (NSCLC), which is the main cause of cancer-related mortality. Moreover, the authors provide insights for developing EVs-based diagnostic, prognostic, and therapeutic.

The role of EVs in modulating cancer cell dormancy is described in the review of (D’Antonio et al.). In this review, the authors elucidate how EVs influence tumor cell dormancy and awakening, focusing on their interaction with tumor and non-tumor cells in primary tumors and pre-metastatic niches. In particular, EV-associated nucleic acids are highlighted as potential targets for diagnostic, prognostic, and therapeutic strategies were also clearly described. Since the awakening of dormant tumor cells contributes to tumor recurrence, this review addresses the potential application of EVs as biomarkers and possible target to prevent cancer relapse and metastasis.

Hepatocellular carcinoma (HCC) is an aggressive cancer with high recurrence and resistance to chemotherapy. The review by Tian et al. illustrated how small EVs from cancer, stromal, and immune cells activate signaling pathways in recipient cells, enhancing stemness properties through the transfer of bioactive molecules during HCC development. As such, the identification of strategies for targeting EV-mediated communication could offer new therapeutic options for HCC.

Finally, the research paper from Yaghjyan et al. examined the relationship between reproductive factors and other breast cancer risk factors with the expression of breast CSC markers (CD44, CD24, and ALDH1A1) in benign breast tissue samples from 439 cancer-free women in the Nurses’ Health Study. Results showed that factors such as the length of time between menarche and first birth, time since last pregnancy, and duration of breastfeeding were inversely associated with CD44 and/or ALDH1A1 expression in breast epithelium and stroma. These findings suggest a link between reproductive factors and CSC marker levels in benign breast tissue, thus underlying a potential link to breast cancer risk.

Overall, this Research Topic underscores the importance of EVs as master regulators of tumor cells and stroma/immune cell reprogramming. This EV-mediated cross-talk also allows cancer cells to acquire stem cell-like characteristics, increasing their potential to fuel the primary tumor and spread to distant organs to initiate metastasis. As reviewed in this Research Topic for different tumor types, EVs represent potential therapeutic targets to impair this vicious interaction between cancer cells and the tumor microenvironment. that maintains and nourishes tumors. Finally, EVs can also act as functional biomarkers of clinical outcomes, paving the way for their use in guiding cancer management.
